# The relationship between sepsis bundle delay intervals and in-hospital mortality of sepsis patients in ICU

**DOI:** 10.3389/fmed.2026.1756833

**Published:** 2026-08-11

**Authors:** Chenhong Zhang, Hao Huang, Xiaosheng Zhu, Weiwei Tang, Lei Cao, Qiuming Bao, Linfeng Zhao, Chun Chen, Zhaoqing Bai

**Affiliations:** Department of Emergency, Anqing Medical Center, Anhui Medical University, Anqing, Anhui, China

**Keywords:** in-hospital mortality, recognition delay, sepsis, sepsis bundle therapy, treatment delay

## Abstract

**Purpose:**

Early sepsis bundle therapy serves as the cornerstone in the management of sepsis, yet the optimal time targets for its administration remain unclear. We quantified the time interval for the use of bundled therapy and assessed the association between the delay in initiating bundled therapy and in-hospital mortality among patients with suspected sepsis treated in the ED.

**Materials and methods:**

Patients with sepsis were collected retrospectively from January 2019 to December 2024, and we divided the sepsis bundle therapy time into two intervals: ED to sepsis bundle orders (recognition delay) and sepsis bundle orders to sepsis bundle implementation (treatment delay). Subsequent statistical analyses were conducted to evaluate the correlations between these two time intervals and patient prognosis.

**Results:**

After adjusting for confounding factors, the two time intervals were associated with the mortality rate during hospitalization, but this association was observed in longer delays compared to the no-delay control group.

**Conclusion:**

The timing of initiating sepsis bundle therapy should be highlighted by medical professionals, and additional investigations are required to refine the optimal timing for the clinical implementation of bundle therapy.

## 1 Introduction

Sepsis is a serious global health problem, mainly because the host’s dysregulation of the inflammatory response leads to organ dysfunction. Despite advancements in current sepsis management, its mortality rate remains persistently high. According to statistical data from 2017, there were 48.9 million cases of sepsis worldwide, resulting in approximately 11 million deaths, accounting for nearly 19.7% of global mortality. A substantial number of survivors experienced various organ function impairments (1). First introduced in 2004, the “sepsis bundle” has stood as a core component of the Surviving Sepsis Campaign (SSC) and played a pivotal role in guiding the revision and refinement of SSC guidelines. Following the publication of the Surviving Sepsis Campaign: International Guidelines for Management of Sepsis and Septic Shock: 2016, the “hour-1 bundle” for sepsis management was introduced. This bundle comprises lactate measurement, blood culture collection, administration of broad-spectrum antibiotics, and infusion of 30 mL/kg crystalloid fluid in cases of hypotension or lactate level ≥4 mmol/L. The guideline released in 2018 indicates that this kind of bundled treatment should be initiated within 1 h after the patient is triaged at the ED or, if referred from another care location, from the earliest chart annotation consistent with all elements of sepsis (formerly severe sepsis) or septic shock ascertained through chart review (2). This requirement aims to shorten the clinical treatment latency and deliver more efficient care to patients with sepsis.

Since the establishment of the “sepsis bundle” for sepsis treatment, controversies have persisted. In the 2018 updated guidelines, it is recommended that all components of the sepsis bundle be initiated within 1 hour of sepsis identification. Consequently, this approach has been linked to a substantial reduction in patient mortality and a marked decrease in hospital length of stay, thereby enhancing the overall prognosis of patients with sepsis (2). Furthermore, timely administration of antibiotics within 1 h improves prognosis in sepsis patients, whereas each 1-h delay in antibiotic therapy elevates mortality risk. Notably, early fluid resuscitation does not effectively enhance patient outcomes (3–5). Therefore, with the advancement of research, an increasing number of scholars have found that in routine clinical practice, it is challenging to complete all bundled treatments within 1 hour. First, in accordance with the definition of Sepsis-3, the rapid diagnosis of sepsis remains a daunting task (6). Second, bacterial cultures will take between 24 and 48 h to give meaningful results and do not help to make decisions as to whether to start antibiotics. Third, due to regional differences and the uneven distribution of medical resources, not all patients can be diagnosed quickly within an hour after being diagnosed. Furthermore, the compliance rate of 1-h bundle therapy remains relatively low. According to a report from the U.S. Centers for Medicare & Medicaid Services (CMS), the compliance rate of the 1-h bundle varies drastically across different hospitals, ranging from 0 to 100%. A study involving 22 Asian countries and regions reported that the compliance rate of 1-h bundle was 21.5% (7, 8). Furthermore, it is not clear whether the time interval from ED triage to the implementation of the bundle therapy is the optimal measurement interval. Therefore, there is an ongoing debate both domestically and internationally concerning the optimal timing for initiating bundle therapy, with insufficient evidence to reach a consensus. As a result, some studies have suggested the use of 3-h bundle therapy (9). This study primarily quantified the time intervals from ED to the initiation of bundle treatment for patients with suspected sepsis in the ED, and further explored the impact of such time delays on the in-hospital mortality of sepsis patients. By reviewing relevant domestic and international literature, it is found that there is currently a relative paucity of research focusing on the effect of delayed initiation of bundle therapy on the prognosis of sepsis patients.

## 2 Methods

### 2.1 Patients selection

Patients who presented to the ED of our hospital between January 2019 and December 2024 and were subsequently admitted to the intensive care unit (ICU) were retrospectively enrolled. According to the SEPSIS-3 diagnostic criteria: Inclusion criteria included suspicion of infection and at least one of the following severity criteria: serum lactate >2 mmol/L, hypotension with systolic blood pressure <90 mmHg or evidence of organ dysfunction with a SOFA score ≥2 (10). Patients meeting any of the following conditions were excluded: ①aged <18 years;②complicated with severe liver and kidney diseases, advanced malignant tumors, acute heart failure, hematological disorders (including malignant hematological neoplasms, bone marrow failure diseases, hereditary/acquired coagulation and platelet disorders, severe hemolytic diseases, etc.), severe heart diseases, acute cerebrovascular diseases, or with an expected survival time of < 6 months;③suffering from cardiac and respiratory arrest on admission, requiring cardiopulmonary resuscitation and tracheal intubation for rescue;④incomplete clinical data;⑤ICU length of stay ≤24 h (either admitted and discharged within 24 h);⑥failure to obtain informed consent from themselves or their legal representatives;⑦failure to meet the SEPSIS-3 diagnostic criteria for sepsis;⑧pregnant or lactating women. Acute heart failure was defined as the presence of clinical manifestations of acute heart failure, accompanied by either elevated brain natriuretic peptide levels or signs of pulmonary edema on chest imaging. All data included in this study were obtained with the informed consent of patients. All methods were performed in accordance with the relevant guidelines and regulations (Declaration of Helsinki).

### 2.2 Study design

**Figure 1:**
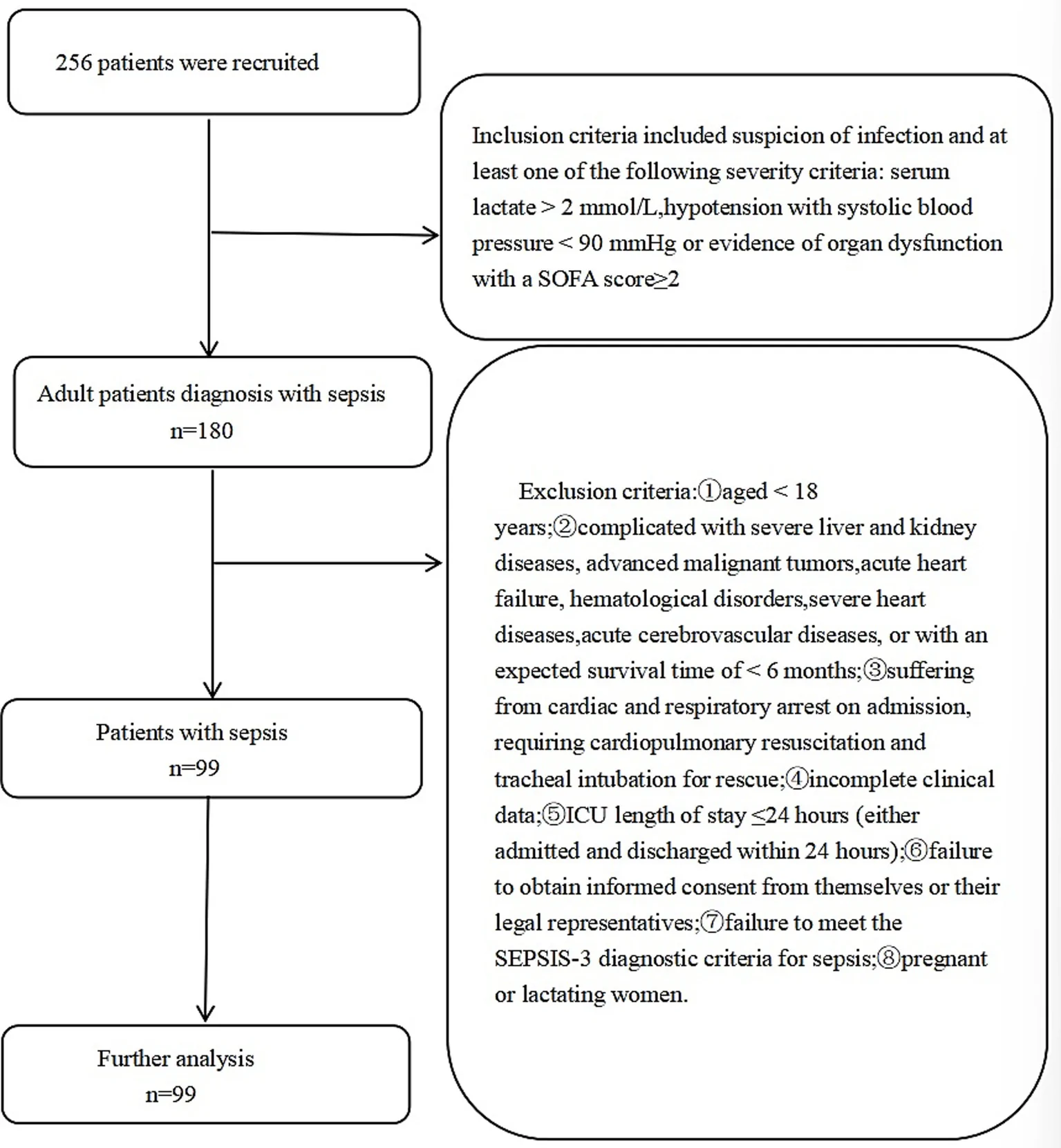
Flowchart for patient selection.

This study was a retrospective investigation conducted on patients with suspected sepsis who presented to the ED of our hospital between January 2019 and December 2024. We collected the total time elapsed from ED presentation to the implementation of the “sepsis bundle” and divided this duration into two intervals. The first interval was defined as the recognition delay time (T1), which refers to the period from the patient’s arrival at the ED to the issuance of orders for the sepsis bundle. The second interval, termed treatment delay time(T2), was the time from the issuance of sepsis bundle orders to the initiation of corresponding treatment measures. The primary outcome measure was in-hospital mortality. In addition, general clinical data of the patients were collected. Secondary outcomes included the development of acute heart failure within 24 h, length of ICU stay, duration of mechanical ventilation during hospitalization, and duration of renal replacement therapy. The detailed study process is illustrated in the flowchart (Figure 1). All the aforementioned data were extracted from the hospital’s electronic information system.

### 2.3 Statistical analysis

Statistical analyses were performed using SPSS 27.0 software. Variables with normal distribution were expressed as mean (standard deviation, SD), continuous variables with non-normal distribution were expressed as median (interquartile range, IQR), and categorical variables were expressed as percentages. Generalized linear mixed models (GLMMs) were employed to evaluate the effects of delay delay and treatment delay on the primary and secondary outcomes.

## 3 Results

### 3.1 Participant characteristics

Table 1 summarizes the demographic and clinical characteristics of 99 patients who met the study’s inclusion criteria. Among these patients, 57 were male (57.58%) and 42 were female (42.42%). The cohort included 46 patients with septic shock (46.46%) and 53 patients without septic shock (53.54%). During the study follow-up period, 55 patients (55.56%) died from their underlying conditions. Pulmonary infection was the most common underlying disease, affecting 57 patients (57.58%). The predominant comorbidities observed in the study population included respiratory failure (40.40%), renal insufficiency (50.51%), hepatic dysfunction (31.31%), heart dysfunction (25.25%), and hypertension (20.20%).

**Table 1:** The general characteristics of patients.

Variables	Values
Sex (*n*, %)	
Male	57 (57.58%)
Female	42 (42.42%)
Age(years)	63.78 ± 15.53
PLT (×10^9/L)	123.00 (51.00, 216.00)
Lactic acid (mmol/L)	3.10 (1.57, 5.73)
Creatinine (umol/L)	145.00 (107.00, 244.00)
CRP (mg/L)	102.00 (38.80, 247.00)
PCT (ng/mL)	10.42 (2.10, 57.77)
SOFA score on the first day	5.00 (3.00, 8.00)
Septic shock	46 (46.46%)
Primary disease, *n* (%)	
Chest infection	57 (57.58%)
Nervous system infection	2 (2.02%)
Genitourinary infection	13 (13.13%)
Soft tissue infection	2 (2.02%)
Abdominal cavity infection	8 (8.08%)
Mixed infection	1 (1.01%)
Skin infection	2 (2.02%)
Blood infection	11 (11.11%)
Intestinal infection	2 (2.02%)
Pulmonary infection	1 (1.01%)
Complications, *n* (%)	
Respiratory failure	40 (40.40%)
Renal insufficiency	50 (50.51%)
Hepatic dysfunction	31 (31.31%)
Gastrointestinal bleeding	10 (10.10%)
Heart failure	25 (25.25%)
Hypertension	20 (20.20%)
Cerebral hemorrhage	1 (1.01%)
Primary endpoint	
In-hospital mortality rate, *n* (%)	55 (55.56%)
Secondary endpoints	
Acute heart failure within 24 h, *n* (%)	33 (33.33%)
Intensive care unit, *n* (%)	99 (100.00%)
Mechanical ventilation, *n* (%)	67 (67.68%)
Renal replacement therapy, *n* (%)	14 (14.14%)
T1	
<1 h	10 (10.10%)
1–3 h	39 (39.39%)
3.01–6 h	22 (22.22%)
>6 h	28 (28.28%)
T2	
0–0.5 h	9 (9.09%)
0.51–1 h	6 (6.06%)
1.01–2 h	12 (12.12%)
2.01–3 h	19 (19.19%)
>3 h	53 (53.54%)

### 3.2 Effect of recognition delay time and treatment delay time on in-hospital mortality

Table 2 demonstrates the impacts of T1 and T2 on in-hospital mortality outcomes. When T1 > 6 h, the risk of in-hospital death increased significantly (OR = 3.215, *p* < 0.05). Similarly, a prolonged T2 (beyond 1 h) was associated with a marked elevation in mortality risk, and this risk intensified with the extension of delay time. Particularly, when T2 exceeded 3 h, the mortality risk reached its peak (OR = 4.117, *p* < 0.001). These associations were analyzed after adjusting for potential confounding factors, including PLT, lactic acid, creatinine, CRP, PCT, SOFA, primary disease and complications.

**Table 2:** The impact of T1 and T2 on mortality outcomes.

Time	Group	OR	95% CI	p
T1	<1 h	1	—	—
	1–3 h	1.127	0.845–1.503	0.423
	3–6 h	1.384	0.972–1.970	0.071
	>6 h	3.215	2.104–4.912	<0.001
T2	<0.5 h	1	—	—
	0.51–1 h	1.245	0.937–1.655	0.132
	1.01–2 h	1.572	1.102–2.241	0.012
	2.01–3 h	2.894	1.863–4.497	<0.001
	>3 h	4.117	2.548–6.653	<0.001

### 3.3 Effect of recognition delay time and treatment delay time on acute heart failure within 24 hours

Table 3 presents the effects of T1 and T2 on acute heart failure within 24 h after symptom onset. T1 > 6 h significantly increased the risk (OR = 2.764, *p* < 0.001), while there was no significant difference within 1–6 h. For T2, the risk of acute heart failure gradually increased after 1 h of delay, with the highest risk noted in cases with T2 exceeding 3 h (OR = 3.028, *p* < 0.001). These results were adjusted for potential confounding factors, including PLT, lactic acid, creatinine, CRP, PCT, SOFA, primary disease and complications.

**Table 3:** The impact of T1 and T2 on 24-h acute heart failure.

Time	Group	OR	95% CI	*p*
T1	<1 h	1	—	—
	1–3 h	1.085	0.792–1.485	0.612
	3–6 h	1.327	0.925–1.904	0.125
	>6 h	2.764	1.842–4.147	<0.001
T2	<0.5 h	1	—	—
	0.51–1 h	1.172	0.879–1.562	0.279
	1.01–2 h	1.435	1.003–2.053	0.048
	2.01–3 h	2.105	1.387–3.195	0.001
	>3 h	3.028	1.912–4.797	<0.001

### 3.4 Effect of recognition delay time and treatment delay time on ICU stay duration

Table 4 presents the influence of T1 and T2 on the duration of ICU stay. For T1, a delay exceeding 6 h significantly prolonged the ICU stay (OR = 2.341, *p* < 0.001). In contrast, T1 ranging from 1 to 6 h showed no statistically significant difference in ICU stay duration compared with the reference group. Regarding T2, a delay of more than 2 h also led to a significant extension of ICU stay; notably, the prolongation effect was most pronounced when T2 exceeded 3 h (OR = 2.875, *p* < 0.001). After controlling for confounding factors (PLT, lactic acid, creatinine, CRP, PCT, SOFA, primary disease and complications).

**Table 4:** The impact of T1 and T2 on ICU days.

Time	Group	OR	95% CI	*p*
T1	<1 h	1	—	—
	1–3 h	1.043	0.775–1.404	0.784
	3–6 h	1.215	0.857–1.722	0.273
	>6 h	2.341	1.578–3.472	<0.001
T2	<0.5 h	1	—	—
	0.51–1 h	1.158	0.863–1.554	0.329
	1.01–2 h	1.384	0.971–1.972	0.073
	2.01–3 h	2.017	1.325–3.071	0.001
	>3 h	2.875	1.802–4.586	<0.001

### 3.5 Effect of recognition delay time and treatment delay time on mechanical ventilation

Table 5 was used to assess the influence of T1 and treatment delay time T2 on the duration of mechanical ventilation. The results indicated that a diagnosis delay exceeding 6 h significantly prolonged the duration of mechanical ventilation (OR = 2.518, *p* < 0.001), while there was no significant difference within 1–6 h. T2 showed a gradual increase in mechanical ventilation duration after > 1 h, and the impact was the greatest when it exceeded 3 h (OR = 3.164, *p* < 0.001). These results were obtained after adjusting for potential confounding factors, including PLT, lactic acid, creatinine, CRP, PCT, SOFA, primary disease and complications.

**Table 5:** The impact of T1 and T2 on mechanical ventilation time.

Time	Group	OR	95% CI	*p*
T1	<1 h	1	—	—
	1–3 h	1.092	0.802–1.486	0.575
	3–6 h	1.254	0.879–1.789	0.214
	>6 h	2.518	1.694–3.743	<0.001
T2	<0.5 h	1	—	—
	0.51–1 h	1.203	0.901–1.606	0.210
	1.01–2 h	1.463	1.025–2.088	0.036
	2.01–3 h	2.231	1.473–3.379	<0.001
	>3 h	3.164	1.996–5.016	<0.001

### 3.6 Effect of recognition delay time and treatment delay time on the duration of renal replacement therapy

Table 6 was used to analyze the influencing factors of renal replacement therapy duration. The results showed that when the T1 exceeded 6 h, the duration of renal replacement was significantly prolonged (OR = 2.657, *p* < 0.001), whereas no statistically significant difference was observed in the T1 range of 1–6 h. T2 showed a significant increase in the number of renal replacement days after exceeding 2 h, especially when it exceeded 3 h, with the greatest impact (OR = 2.747, *p* < 0.001). These results were obtained after adjusting for potential confounding factors, including PLT, lactic acid, creatinine, CRP, PCT, SOFA, primary disease and complications.

**Table 6:** The impact of T1 and T2 on renal replacement therapy.

Time	Group	OR	95% CI	*p*
T1	<1 h	1	—	—
	1–3 h	1.065	0.777–1.460	0.699
	3–6 h	1.182	0.828–1.687	0.356
	>6 h	2.657	1.789–3.947	<0.001
T2	<0.5 h	1	—	—
	0.51–1 h	1.135	0.848–1.519	0.395
	1.01–2 h	1.327	0.930–1.894	0.120
	2.01–3 h	1.984	1.310–3.005	0.001
	>3 h	2.747	1.732–4.357	<0.001

### 3.7 Effect of recognition delay time and treatment delay time on in-hospital mortality in patients without septic shock

Table 7 (for the non-septic shock group) indicates that the risk of in-hospital death increased significantly when the T1 exceeded 6 h (OR = 3.341, *p* < 0.001), whereas no statistically significant difference was observed in the T1 range of 1 to 6 h. For T2, the risk of death gradually elevated after exceeding 1 h, reaching the peak when T2 surpassed 3 h (OR = 4.298, *p* < 0.001). The above results were adjusted for potential confounding factors, including PLT, lactic acid, creatinine, CRP, PCT, SOFA, primary disease and complications.

**Table 7:** The impact of T1 and T2 on mortality outcomes without septic shock.

Time	Group	OR	95%CI	P
T1	<1 h	1	—	—
	1–3 h	1.156	0.892–1.498	0.387
	3–6 h	1.428	1.012–2.015	0.063
	>6 h	3.341	2.245–4.972	<0.001
T2	<0.5 h	1	—	—
	0.51–1 h	1.312	0.982–1.752	0.118
	1.01–2 h	1.645	1.152–2.348	0.009
	2.01–3 h	3.012	1.935–4.689	<0.001
	>3 h	4.298	2.678–6.898	<0.001

### 3.8 Effect of recognition delay time and treatment delay time on secondary endpoints in patients without septic shock

As presented in Tables 8–11, when the T1 exceeded 6 h, it significantly elevated the risk of acute heart failure within 24 h (OR = 2.892, *p* < 0.001), remarkably prolongs the length of ICU stay (OR = 2.458, *p* < 0.001) and the duration of mechanical ventilation (OR = 2.645, *p* < 0.001), and notably increases the number of days of renal replacement therapy (OR = 2.785, *p* < 0.001). In contrast, no significant differences in the aforementioned secondary outcomes were observed when T1 ranged from 1 to 6 h. Meanwhile, the results also indicate that when the T2 exceeded 1 h, the risk of acute heart failure within 24 h and the duration of mechanical ventilation both showed a gradual upward trend, with the most pronounced effects detected when T2 exceeded 3 h (OR = 3.187, *p* < 0.001; OR = 3.328, *p* < 0.001). When T2 exceeded 2 h, the length of ICU stay was significantly prolonged and the duration of renal replacement therapy increased markedly, with the most pronounced impact observed when T2 exceeded 3 h (OR = 3.025, *p* < 0.001; OR = 2.892, *p* < 0.001). These associations were further analyzed after adjusting for potential confounding factors, including PLT, lactic acid, creatinine, CRP, PCT, SOFA, primary disease and complications.

**Table 8:** The impact of T1 and T2 on 24-h acute heart failure without septic shock.

Time	Group	OR	95% CI	*p*
T1	<1 h	1	—	—
	1–3 h	1.124	0.832–1.518	0.587
	3–6 h	1.405	0.975–2.025	0.112
	>6 h	2.892	1.925–4.345	<0.001
T2	<0.5 h	1	—	—
	0.51–1 h	1.231	0.921–1.645	0.254
	1.01–2 h	1.512	1.058–2.161	0.042
	2.01–3 h	2.245	1.478–3.410	<0.001
	>3 h	3.187	2.012–5.049	<0.001

**Table 9:** The impact of T1 and T2 on ICU days without septic shock.

Time	Group	OR	95% CI	*p*
T1	<1 h	1	—	—
	1–3 h	1.087	0.815–1.450	0.752
	3–6 h	1.278	0.902–1.811	0.241
	>6 h	2.458	1.655–3.651	<0.001
T2	<0.5 h	1	—	—
	0.51–1 h	1.215	0.908–1.626	0.302
	1.01–2 h	1.452	1.018–2.071	0.065
	2.01–3 h	2.125	1.397–3.232	<0.001
	>3 h	3.025	1.895–4.827	<0.001

**Table 10:** The impact of T1 and T2 on the mechanical ventilation time without septic shock.

Time	Group	OR	95% CI	*p*
T1	<1 h	1	—	—
	1–3 h	1.145	0.842–1.557	0.538
	3–6 h	1.328	0.931–1.894	0.198
	>6 h	2.645	1.778–3.935	<0.001
T2	<0.5 h	1	—	—
	0.51–1 h	1.265	0.947–1.689	0.187
	1.01–2 h	1.532	1.073–2.188	0.031
	2.01–3 h	2.351	1.552–3.562	<0.001
	>3 h	3.328	2.098–5.279	<0.001

**Table 11:** The impact of T1 and T2 on renal replacement therapy without septic shock.

Time	Group	OR	95% CI	*p*
T1	<1 h	1	—	—
	1–3 h	1.112	0.812–1.523	0.663
	3–6 h	1.245	0.872–1.778	0.321
	>6 h	2.785	1.875–4.137	<0.001
T2	<0.5 h	1	—	—
	0.51–1 h	1.192	0.891–1.595	0.362
	1.01–2 h	1.405	0.985–2.004	0.105
	2.01–3 h	2.087	1.378–3.161	<0.001
	>3 h	2.892	1.822–4.589	<0.001

### 3.9 Effects of recognition delay time and treatment delay time on in-hospital mortality in patients with septic shock

As shown in Table 12 (septic shock group), the effect of T1 exceeding 6 h on in-hospital mortality risk was not statistically significant (*p* = 0.135). In contrast, the mortality risk increased progressively when T2 exceeded 1 h, with the highest risk observed when T2 exceeded 3 h (OR = 3.952, *p* < 0.001). These results were adjusted for potential confounding factors, including PLT, lactic acid, creatinine, CRP, PCT, SOFA, primary disease and complications.

**Table 12:** The impact of T1 and T2 on mortality outcomes with septic shock.

Time	Group	OR	95% CI	*p*
T1	<1 h	1	—	—
	1–3 h	1.098	0.827–1.458	0.521
	3–6 h	1.352	0.952–1.920	0.089
	>6 h	3.087	0.867–4.610	0.135
T2	<0.5 h	1	—	—
	0.51–1 h	1.198	0.899–1.597	0.215
	1.01–2 h	1.501	1.053–2.140	0.025
	2.01–3 h	2.765	1.778–4.298	<0.001
	>3 h	3.952	2.458–6.351	<0.001

### 3.10 Effects of recognition delay time and treatment delay time on secondary endpoints in patients with septic shock

As shown in Tables 13–16 (for the septic shock group), when the T1 exceeded 6 h, there was a significant increase in the risk of acute heart failure within 24 h (OR = 2.685, *p* < 0.001). Additionally, T1 exceeding 6 h remarkably prolonged the length of ICU stay and duration of mechanical ventilation (OR = 2.284, *p* < 0.001; OR = 2.412, *p* < 0.001, respectively) and increased the number of days of renal replacement therapy (OR = 2.512, *p* < 0.001). In contrast, no significant differences in these secondary endpoints were observed when T1 was within the 1–6 h range. Meanwhile, the T2 exerted no significant effects on the incidence of acute heart failure, length of ICU stay, duration of mechanical ventilation, or number of days of renal replacement therapy across all groups (all *p* > 0.05). These results were obtained after adjusting for potential confounding factors, including PLT, lactic acid, creatinine, CRP, PCT, SOFA, primary disease and complications.

**Table 13:** The impact of T1 and T2 on 24-h acute heart failure with septic shock.

Time	Group	OR	95% CI	*p*
T1	<1 h	1	—	—
	1–3 h	1.067	0.788–1.445	0.678
	3–6 h	1.289	0.902–1.842	0.165
	>6 h	2.685	1.792–4.022	<0.001
T2	<0.5 h	1	—	—
	0.51–1 h	1.143	0.857–1.525	0.358
	1.01–2 h	1.397	0.978–1.996	0.066
	2.01–3 h	1.423	0.935–2.165	0.102
	>3 h	1.489	0.982–2.258	0.061

**Table 14:** The impact of T1 and T2 on ICU days with septic shock.

Time	Group	OR	95% CI	*p*
T1	<1 h	1	—	—
	1–3 h	1.021	0.761–1.370	0.887
	3–6 h	1.187	0.837–1.684	0.337
	>6 h	2.284	1.539–3.390	<0.001
T2	<0.5 h	1	—	—
	0.51–1 h	1.121	0.838–1.500	0.441
	1.01–2 h	1.342	0.941–1.914	0.104
	2.01–3 h	1.348	0.887–2.048	0.164
	>3 h	1.385	0.873–2.197	0.167

**Table 15:** The impact of T1 and T2 on the mechanical ventilation time with septic shock.

Time	Group	OR	95% CI	*p*
T1	<1 h	1	—	—
	1–3 h	1.054	0.774–1.435	0.735
	3–6 h	1.215	0.853–1.731	0.281
	>6 h	2.412	1.622–3.587	<0.001
T2	<0.5 h	1	—	—
	0.51–1 h	1.167	0.874–1.559	0.296
	1.01–2 h	1.421	0.996–2.027	0.052
	2.01–3 h	2.157	0.842–3.270	0.109
	>3 h	3.052	0.925–4.838	0.096

**Table 16:** The impact of T1 and T2 on renal replacement therapy with septic shock.

Time	Group	OR	95% CI	*p*
T1	<1 h	1	—	—
	1–3 h	1.042	0.761–1.427	0.798
	3–6 h	1.158	0.812–1.651	0.417
	>6 h	2.512	1.689–3.736	<0.001
T2	<0.5 h	1	—	—
	0.51–1 h	1.098	0.821–1.468	0.536
	1.01–2 h	1.289	0.903–1.840	0.163
	2.01–3 h	1.402	0.923–2.129	0.113
	>3 h	1.572	0.984–2.512	0.058

## 4 Discussion

Early administration of sepsis bundle therapy constitutes the cornerstone of care for patients with sepsis. However, the specific time targets for sepsis bundle implementation remain unclear, and there is no consensus on which time intervals should be monitored or prioritized for minimization. To better elucidate the relationship between the timing of sepsis bundle therapy and patient outcomes, we examined two key time intervals: the duration from a patient’s arrival at the ED to the issuance of sepsis bundle orders (recognition delay time, T1), and the interval from order issuance to the initiation of sepsis bundle treatment measures (treatment delay time, T2). In clinical practice, treatment delays are prevalent; however, the majority of the total time is attributed to delays in recognition. This study quantified the time intervals of sepsis bundle therapy and investigated the impact of delayed implementation of these intervals on the prognosis of patients with suspected sepsis in the ED. In clinical practice, relatively few patients are able to receive bundled treatment within 1 hour. Historically, primary attention has been focused on treatment delays—these delays are not only associated with increased mortality but also represent a straightforward measure that can be readily optimized in clinical settings.

In our study, treatment delay was significantly associated with in-hospital mortality among sepsis patients, as well as secondary outcomes including acute heart failure within 24 h, ICU length of stay, duration of mechanical ventilation, and days of renal replacement therapy. However, the results of this study indicated that there might be a time threshold for the impact of treatment delay on the prognosis of patients with sepsis. Data showed that an association was observed when the delay time exceeded 3 h. In contrast, no statistically significant difference was observed in the samples with a shorter delay time (<1 h), but this did not completely rule out its potential clinical impact.

Recognition delay time was also significantly associated with in-hospital mortality and the occurrence of secondary outcomes. Compared with a recognition delay of less than 1 h, a delay exceeding 6 h was associated with an increase in mortality rate, along with an elevated risk of secondary adverse outcomes. Therefore, this finding also raised our consideration: simply focusing on treatment delay might not fully reflect the complexity of clinical timing. Notably, among patients with septic shock, treatment delay time was the sole interval significantly associated with in-hospital mortality. This may be attributed to the more severe clinical condition of septic shock patients, which facilitates their prompt identification in clinical practice.

Previous studies have suggested that interventions such as early administration of antibiotics and lactate measurement within 1 hour may improve the prognosis of sepsis patients; however, this remains a topic of intense debate. To date, no conclusive evidence has demonstrated additional benefits of performing fluid resuscitation within 1 hour (5). Over the past few years, the SSC has issued multiple recommendations with varying time and target goals. None of these studies have established that a specific time target is more effective than others in improving patient outcomes, leading to persistent controversy regarding the initiation and completion timelines of the sepsis bundle (11, 12).

Our study indicated that there might be a threshold for the impact of treatment delay and recognition delay on patients with sepsis. Although previous guidelines emphasized the importance of early intervention, our data showed that completing all evaluations and treatments within 1 h might not bring universal additional benefits. These findings prompted several reflections: strict compliance with the “zero-tolerance” 1-h window for all patients with suspected sepsis might not be the only effective strategy, and it could have led to adverse consequences—such as the development of antibiotic resistance in the ICU and cardiac or other organ dysfunction. All clinicians should take this into account. When managing patients with suspected sepsis in clinical practice, clinicians may need to more cautiously consider the necessity of strictly adhering to the “1-h” time window. This does not negate the value of early treatment, but suggests that clinicians should seek a balance between pursuing speed and ensuring diagnostic accuracy to avoid potential risks such as antibiotic abuse or increased organ function burden that may be caused by excessive pursuit of timeliness. Additionally, it may be clinically rational to extend the time window for diagnostic evaluation before the administration of bundle therapy in such patients.

This study had several limitations, and our findings must therefore be interpreted with the utmost caution. First, the present investigation was a single-center, retrospective study with a small sample size (*n* = 99). This study design substantially limited the statistical power and generalizability of our results, a limitation that was particularly pronounced in subgroup analyses (e.g., septic shock vs. non-shock) due to inadequate statistical power. While our findings may offer valuable insights for optimizing bundle therapy in patients with sepsis moving forward, large-scale prospective studies are warranted to further validate the conclusions of this paper. Second, selection bias was a key limitation. Our study cohort was restricted to patients admitted to the ICU, excluding less severe sepsis cases managed in general wards or the ED. This consequently restricted the applicability of our results to the broader population of patients with sepsis presenting to the emergency department. Third, concerns persisted regarding measurement accuracy and residual confounding. Our definition of “recognition delay” was based on timestamped order entries in the electronic medical record system, which may not accurately reflect the actual moment clinicians first developed clinical suspicion or established the diagnosis—thus introducing potential documentation bias. Furthermore, we were unable to control for several key clinical variables that may have had a substantial impact on study outcomes, including illness severity beyond baseline SOFA score trends, the timing of source control, the appropriateness of antibiotics, and the type and volume of fluids administered—none of which were analyzed in this study. These unmeasured variables constituted significant residual confounders. Therefore,future efforts should focus on conducting prospective, multicenter studies with large sample sizes. These studies should incorporate more relevant indicators and expand the geographical scope and population coverage to help reduce bias. Given these limitations, the conclusions derived from this study were necessarily preliminary and intended for clinical reference only. We do not advocate for the abandonment of current clinical guidelines by clinicians. Instead, our data should be viewed as hypothesis-generating, highlighting the need for large-scale prospective investigations to determine whether a strict 1-h treatment target is optimal for all patients with sepsis, or whether a more individualized, flexible approach based on distinct patient clinical trajectories may yield greater benefits.

## 5 Conclusion

In summary, although delays in sepsis recognition and treatment were both critical determinants of patient outcomes, strict adherence to time-based bundle therapy targets in clinical practice was complicated by factors including variability in clinical settings and the inherent complexity of sepsis pathophysiology. We strongly emphasized that these results were constrained by the study’s retrospective design and small sample size, and they should not be used to revise current clinical practice guidelines, which are supported by a far more robust evidence base. Instead, this study called for further research to refine the timing of sepsis bundle therapy, ensuring that the pursuit of rapid intervention did not compromise diagnostic accuracy or exacerbate the emergence of antibiotic resistance. Clinicians should continue to remain attentive to the ongoing discourse surrounding optimal time-based targets for sepsis bundle initiation.

## Data Availability

The original contributions presented in the study are included in the article/supplementary material, further inquiries can be directed to the corresponding authors.
